# The role and implication of platelet‐rich plasma in male factor infertility: A systematic review of human studies

**DOI:** 10.1111/andr.70048

**Published:** 2025-04-17

**Authors:** Karl H. Pang

**Affiliations:** ^1^ Division of Surgery and Interventional Science University College London London UK; ^2^ Department of Urology Chelsea and Westminster Hospital NHS Foundation Trust London UK

**Keywords:** cryopreservation, male infertility, platelet‐rich plasma, sperm retrieval

## Abstract

**Background:**

Cryopreservation causes sperm injury and the success of surgical sperm retrieval (SSR) for azoospermic men is just over half depending on the cause of azoospermia. The role of autologous platelet‐rich plasma (PRP) in male factor infertility (MFI) is unclear.

**Objective:**

To conduct a systematic review of the role of PRP in MFI focusing on human studies.

**Methods:**

A systematic review was conducted using PubMed with reference to the PRISMA 2020 statement. The risk of bias assessment of the included studies was performed using the JBI assessment checklists. Outcome measures included the effects of PRP on cryopreservation, semen parameters, and SSR.

**Results:**

The search retrieved 119 articles and 10 met the pre‐defined PICO criteria. These included 7 prospective studies, 1 randomised‐controlled study, 1 retrospective study, and 1 case report. PRP appeared to improve semen parameters, decrease DNA fragmentation, improve recovery of cryopreserved sperm, and improve SSR rate. A case report demonstrated successful intracytoplasmic sperm injection and pregnancy following incubation of semen with PRP in a patient with previous failed IVF attempts.

**Discussion and conclusion:**

This is the first systematic review summarising data from human studies on the role of PRP in MFI. The inclusion criteria and outcomes of across studies varied, limiting the ability to conduct a quantitative analysis. Data from early studies on PRP in MFI are promising. However, there is a lack of well‐designed clinical studies on this topic, and further research is needed to replicate current findings and evaluate the potential benefits of PRP in MFI.

## 1 INTRODUCTION

Assisted reproductive technology (ART) offers a treatment option for male factor infertility (MFI). ART often involves seminal cryopreservation, and for men who are azoospermic or unable to provide a semen sample, surgical sperm retrieval (SSR) becomes necessary.^1^, ^2^ Cryopreservation is not only used in MFI, but also for men experiencing testicular tissue loss due to trauma or orchidectomy, those undergoing cancer treatments like chemoradiotherapy, and individuals transitioning from male to female.^3^ While cryopreservation is a valuable tool, the freeze–thaw process can affect sperm viability, motility, and structure.^4^ The success of ART largely depends on the quality and quantity of sperm retrieved, whether from ejaculates, SSR, or cryopreserved samples, highlighting the need for ongoing research to improve semen analysis, SSR techniques, and cryoprotection methods.^4^, ^5^

Platelet‐rich plasma (PRP), derived from whole blood through centrifugation, contains platelet concentrations at least three times higher than normal. Upon activation, PRP granules release growth factors such as platelet‐derived growth factor (PDGF), epidermal growth factor (EGF), transforming growth factor beta (TGF‐β), insulin‐like growth factor‐1 (IGF‐1), and vascular endothelial growth factor (VEGF), which promote angiogenesis, cell differentiation, proliferation, and tissue remodeling.^6^ Given its regenerative properties, PRP has been explored across a variety of medical fields, including musculoskeletal injuries,^7^ facial ageing,^8^ erectile dysfunction,^9^ Peyronie's disease,^10^ and infertility.^11^

In female factor infertility, PRP has shown potential in enhancing ovarian reserve,^12^ improving implantation,^13^ and supporting fertilisation in ART.^11^ Research into the use of PRP for MFI is still in its early stages. This systematic review aims to summarise the current understanding of PRP's role and potential impact on MFI management.

## 2 METHODS

This systematic review was conducted with reference to the PRISMA 2020 statement (Table S1).^14^ A search was performed on PubMed on 22/09/2024 using the following terms: (PRP) AND (male infertility OR semen OR azoospermia OR teratospermia OR asthenospermia OR oligospermia OR OAT OR spermatogenesis OR SSR OR testicular sperm extraction OR TESE OR mTESE) NOT review. A population (P), intervention (I), comparator (C), and outcome (O) framework was applied to define the inclusion criteria: P, adult men with a semen analysis or testicular tissue sampling; I, PRP; C, any treatment comparisons including supplements or placebo; O, semen parameters, spermatogenesis, SSR rate, ART success or pregnancy. Animal studies and reviews were excluded. The search was repeated on 9 February 2025.

Data extracted included the study design, inclusion characteristics, number of patients, PRP preparation (e.g., Kit, centrifuge settings, concentration and volume), injection route, (e.g. into the testicles or semen culture media), post‐PRP semen parameters, histology (spermatogenesis), SSR and pregnancy rate. “Normal seminal” parameters are referred to as seminal parameters higher than the 5th percentile of the WHO population.^3^, ^15^

The risk of bias (RoB) of individual studies was assessed using the JBI assessment tool for RCT, cohort studies or case reports.^16^, ^17^

## 3 RESULTS

### 3.1 Evidence synthesis

**FIGURE 1:**
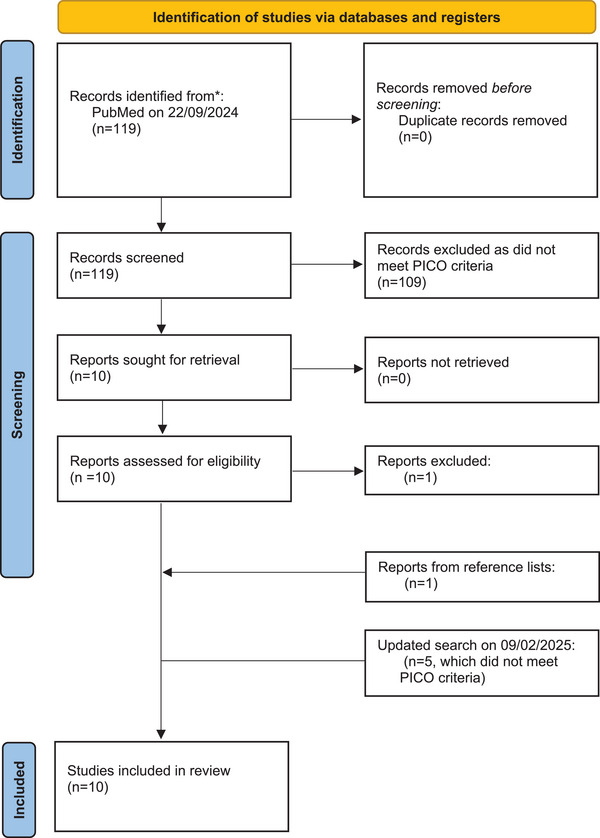
PRISMA 2020 flowchart for the current systematic review.

The initial search (22 September 2024) retrieved 119 articles (Figure 1). After abstract and full‐text screening, nine studies met the pre‐defined PICO criteria. An additional study was included after reviewing the reference lists of the included articles. The updated search on 9 February 2025 identified five new articles, none of which met the PICO criteria. In total, 10 studies were included for analysis, comprising one randomised‐controlled trial (RCT),^18^ seven prospective studies,^19^, ^20^, ^21^, ^22^, ^23^, ^24^, ^25^ one retrospective study^26^ and one case report.^27^ The baseline characteristics and outcomes of each study are summarised in Tables 1 and 2. The results of the RoB assessment of the included studies are presented in Table S2.

**TABLE 1:** Characteristics of included studies.

			Sample size			PRP				
Author	Study design	Inclusion characteristics	Case	Control	Age (years)	Preparation kit	Vol. whole blood	Centrifuge	Concentration	Injection route
**Cryopreservation**										
Lorian et al. 2024^20^	Prospective	Cryopreserved OAT semen	20	20 (no additive)	20–40	Rooyagen,Tehran, Iran	8.5 mL	1600 g, 10 min then 3500 g, 5 min	1×10^5^, 0.5×10^5^ and 0.25×10^5^ per µL	Cryopreserved semen
Nabavinia et al. 2023^21^	Prospective	Cryopreserved normal semen	20	20 (no PRP)	20–30	Rooyagen,Tehran, Iran	8.5 mL volunteer (non‐autologous)	1600 g, 10 min then 3500 g, 5 min	1×10^5^, 0.5×10^5^ and 0.25×10^5^ per µL	Cryopreserved semen
Mirzaei et al. 2022^23^	Prospective	Cryopreserved normal semen	25	25 (0% PRGF)	Mean (SD) 34.9 (4.4)	NR	NR	580 g, 8 min	0, 1%, 5%, 10%	Cryopreserved semen
Yan et al. 2021^24^	Prospective	Cryopreserved normal semen	12	12 (0% PRP)	NR	NR	15 mL	300 g, 10 min then 600 g, 10 min	0, 2%, 5%, 10%	Cryopreserved semen
**Semen and tissue incubation**										
Ulhe et al. 2024^27^	Case	AT semen and 2× failed IVF	1	No	45	NR	10 mL	NR	2%	Semen‐ incubated for 1h
Bader et al. 2020^25^	Prospective	H_2_O_2_‐induced oxidative stress normal semen	30	30 (0% PRP)	Mean (SD) 35 (5)	NR	NR	380 g, 15 min then 1300 g, 8 min	2%, 5%, 10%	H_2_O_2_‐treated semen—incubated for 24h
Salem et al. 2023^22^	Prospective	Human testes from brain‐dead organ donors	4	4	16–28	NR	NR	NR	*Non‐autologous* 1% PRP	Testicular SSCs cultured in DTM for 4 w
**Intratesticular injection**										
Gudelci et al. 2024^19^	Prospective	NOA and failed mTESE	135	No	Mean (SD) 35.4 (5.2)	T‐lab autologous	20 mL	830 g, 8 min	2 mL	Intratesticular 2 mL: upper, mid, lower pole
Fazli et al. 2024^18^	RCT	Severe OAT (sperm count ≤4×10^6^/mL, progressive motility sperm ≤30%, morphologically normal sperm ≤1%)	44	44	NR	NR	5 mL	3000 rpm, 5 min then 3500 rpm 15 min, incubation at 37°C 15 min with 10% calcium chloride to activate PRP. Then 4000 rpm, 10 min	NR	Intratesticular 1–2 mL
Al‐Nasser et al. 2018^26^	Retrospective	NOA	71	No	Mean (SD) 37.6 (6.7)	Dr. PRP kits and equipment, USA	NR	NR	NR	Intratesticular 1/2 mL

*Note*: Normal seminal parameters are referred to as seminal parameters higher than the 5th percentile of the WHO population.

Abbreviations: AT, astheno‐teratospermia; DTM, decellularised testicular matrix; NOA, non‐obstructive azoospermia; NR, not reported; OAT, oligoasthenoteratospermia; PRGF, platelet‐rich growth factor; PRP, platelet‐rich plasma; RCS, randomised‐controlled study; RCT, randomised‐controlled trial; SSC, spermatogonial stem cell.

**TABLE 2:** Role and outcomes of platelet‐rich plasma.

Author	Hormonal	Semen analysis	Oxidative stress, DNA integrity and other assessments	Testicular tissue samples	SSR rate	ART	Pregnancy rate	Conclusion
**Cryopreservation**								
Lorian et al. 2024^20^	NR	20d FT semen: increased progressive motility (*p* < 0.001); increased viability (*p* < 0.001); no improvement in sperm count, non‐progressive motility, normal morphology;	Decreased fragmented DNA (*p* < 0.001); decreased protamine deficiency (*p* < 0.001); no increase in acrosome integrity; decreased MDA (marker of oxidative stress) levels (*p* < 0.001)	n/a	n/a	n/a	n/a	PRP can preserve some sperm parameters and improve DNA fragmentation, protamine deficiency and MDA level in OAT samples.
Nabavinia et al. 2023^21^	NR	20d FT semen: With 1×105 per µL: no increase in sperm count; Increased sperm viability (*p* < 0.04); increased progressive motility (*p* < 0.01); Decreased non‐progressive motility (< 0.05); No decrease in immotile sperm	Increased Chromomycin A3 with 0.25×10^5^ per µL (chromatin integrity deficiency) (*p* < 0.05); decreased DNA fragmentation with 1×10^5^ per µL	n/a	n/a	n/a	n/a	PRP had protective effect on human sperm quality at an optimised concentration.
Mirzaei et al. 2022^23^	NR	FT semen with 1% PRGF vs. control: increased in progressive and total motility; increased in viability; increased in normal morphology; increased in intact acrosome; increased in chromatin structure and integrity	Increased in non‐denatured and non‐fragmented DNA	n/a	n/a	n/a	n/a	PRGF can preserve sperm parameters including motility, viability, morphology, and the integrity of acrosome and DNA during cryopreservation.
Yan et al. 2021^24^	NR	2 w: increased in sperm motility and vitality with 5% PRP (*p* < 0.05)	2 w: improved membrane integrity with 5% PRP (*p* < 0.05); No significant improvement in DNA fragmentation or ROS	n/a	n/a	n/a	n/a	PRP has a partial protective effect on cryopreservation of human spermatozoa.
**Semen and tissue incubation**								
Ulhe et al. 2024^27^	Normal baseline FSH, LH	Improved sperm motility	Decreased DNA fragmentation	n/a	n/a	ICSI rate 100%	100% (positive b‐HCG at day 14)	PRP therapy proved beneficial in improving sperm quality and contributing to the success of frozen embryo transfer.
Bader et al. 2020^25^	NR	24 h: Increased in sperm progressive (*p* < 0.0001) and total motility (*p* < 0.001) with 2% PRP in the non‐stressed group and with 2% (p < 0.0001) and 5% (p < 0.001) PRP in the stressed group; no improvement in normal morphology; increased viability with 2% PRP in non‐stressed (*p* < 0.001) and stressed (*p* < 0.0001) groups; decreased vacuolisation in non‐stressed with 2% (*p* < 0.0001) and 5% (*p* < 0.05) and stressed groups with all concentrations (*p* < 0.0001)	Decrease in ROS‐positive cells and DNA fragmentation in non‐stressed and stressed groups (*p* < 0.05)	n/a	n/a	n/a	n/a	Autologous PRP improves the quality of the sperm, more so in the presence of an H_2_O_2_‐induced oxidative stress.
Salem et al. 2023^22^	NR	NR	30 d: Increased cell viability (*p* = 0.006) 4 w: increased in PRM2 (human SSC‐specific gene); no significant change in other SSC‐genes (PLZF, GFRA1, SCP3); no significant change in apoptosis genes (BAX, BCL‐2)	n/a	n/a	n/a	n/a	DTM supplemented with PRP can provide an effective culture system for the differentiation and viability of SSCs.
**Intratesticular injection**								
Gudelci et al. 2024^19^	No significant difference in pre‐ and post‐PRP serum FSH, LH, total T levels	3 m: azoospermia	NR	Johnson score 2: 61.5%	mTESE 3–9 m post‐PRP: 16.4–27.5% (overall 23.0%)	ICSI rate 86.4%–100% (overall 90.3%)	Live birth: 22.2%–36.8% (overall 50%) per embryo transfer (positive b‐HCG and USS)	Intratesticular PRP injection shows promise as a potential therapeutic approach for NOA patients with prior failed mTESE procedures, demonstrating improved SSR and favourable IVF outcomes
Fazli et al. 2024^18^	NR	3 m: increased in sperm count (*p* = 0.03); increased in motility (*p* = 0.01); no increase in normal morphology (*p* = 0.6)	Decrease in DNA fragmentation (*p* < 0.001)	n/a	n/a	n/a	n/a	PRP effective in increasing sperm concentration and motility and reducing sperm DFI

Al‐Nasser et al. 2018^26^	No significant difference in pre‐ and post‐PRP serum FSH	2–4 m: azoospermia	NR	TESA pre‐PRP, *n* (%) primary and secondary spermatocytes: 50 (54.9) No sperm and no spermatogenesis: 22 (24.2) Few motile and few immotile sperms: 13 (14.3) Oligospermia: 3 (3.3) Other: 3 (3.3)	TESA post‐PRP, *n* (%) primary and secondary spermatocytes: 17 (18.7) No sperm and no spermatogenesis: 20 (22.0) Few motile & few immotile sperms: 27 (29.7) Oligospermia: 2 (2.2) Normal sperm: 2 (2.2) Missing: 23 (25.3)	n/a	n/a	n/a	PRP may have a great potential for the treatment of male infertility and increases spermatogenesis.

Abbreviations: ART, assisted reproductive technology; b‐HCG, beta human chorionic gonadotropin; DFI, DNA fragmentation index; DTM, decellularised testicular matrix; FSH, follicular‐stimulating hormone; FT, frozen‐thawed; ICSI, intracytoplasmic sperm injection; IVF, in vitro fertilisation; LH, luteinising hormone; MDA, malondialdehyde; mTESE, microdissection testicular sperm extraction; n/a, not application; NaCl, sodium chloride; NOA, non‐obstructive azoospermia; NR, not reported; OAT, oligo‐astheno‐teratospermia; PRGF, platelet‐rich growth factor; PRP, platelet‐rich plasma; RCS, randomised‐controlled study; ROS, reactive oxygen species; SD, standard deviation; SSC, spermatogonial stem cell; SSR, surgical sperm retrieval; T, testosterone; TESA, testicular sperm aspiration; USS, ultrasound scan.

PRP has generally been studied in three settings: (1) during cryopreservation, (2) semen incubation, and (3) intratesticular injection. The PRP preparation details are outlined in Table 1, which include the machine used, volume of blood obtained, and the centrifuge settings. Blood volumes ranged from 5 to 20 mL, with centrifuge speeds between 300–1600 g for 5–10 min during the first spin, followed by 600–3500 g for 5–10 min during the second spin. The protocol was typically adhered to the manufacturer guidelines.

### 3.2 Cryopreservation with platelet‐rich plasma

Four prospective comparative studies evaluated the effect of PRP on post‐thaw cryopreserved semen. Adding PRP to cryopreserved semen improved semen parameters and decreased DNA fragmentation in post‐thaw samples compared with controls.^20^, ^21^ This was observed in cryopreserved oligoasthenoteratospermia (OAT) semen (*n* = 20)^20^ and normal semen (*n* = 20).^21^ Yan et al.^24^ demonstrated that 5% PRP supplementation during cryopreservation of normal semen (*n* = 12) also improved sperm motility and vitality after freeze‐thaw at 2 weeks compared to the control (0% PRP) (*p* < 0.05). However, no significant improvements were observed in DNA fragmentation or oxidative stress. Moreover, adding 1% platelet‐rich growth factor (PRGF) during cryopreservation of normal semen (*n* = 25) significantly improved sperm motility, viability, normal morphology and acrosome and chromatin integrity after post‐thaw compared to 0% PRGF. The proportion of non‐denatured and non‐fragmented DNA was also higher compared to the control.^23^

### 3.3 Semen and tissue incubation with platelet‐rich plasma

Two studies assessed the effect of PRP on semen incubation, whilst 1 study analysed PRP's effect on testis tissue incubation. In a case report of a man with asthenoteratospermia and two failed in‐vitro fertilisation (IVF) cycles, semen incubation with PRP improved sperm motility and decreased DNA fragmentation. Subsequently, conception was achieved via intracytoplasmic sperm injection (ICSI).^27^

In a prospective study of 30 men, Bader et al.^25^ examined the effect of PRP on H_2_O_2_‐induced oxidative stress and non‐stressed normal semen. Compared with 0% (control), 2%–5% PRP increased sperm progressive and total motility and viability, whilst decreasing vacuolisation and reactive oxidative species (ROS) and DNA fragmentation in both non‐stressed and stressed groups, with more pronounced effects in the stressed group.

When spermatogonial stem cells (SSC) obtained from testicular tissue of brain‐dead organ donors (*n* = 4) were cultured on human decellularised testicular matrix (DTM) with 1% PRP, cell viability increased after 30 days. In addition, the expression of a spermatogenesis gene marker (PRM2) increased at 4 weeks compared to controls (0% PRP).^22^

### 3.4 Intratesticular injection of platelet‐rich plasma

Three studies evaluated intratesticular PRP injections in men with abnormal semen parameters. Gudelci et al.^19^ analysed the outcomes of intratesticular PRP injection in 135 men with non‐obstructive azoospermia (NOA) and previous unsuccessful microdissection testicular sperm extraction (mTESE). After 3–9 months of PRP injection, the SSR rate was 23.0%, the ICSI fertilisation rate was 90.3% and the pregnancy rate was 36.8%. The SSR rates were highest at 3–4 months following PRP with decreasing rates over time.

A randomised study of 88 infertile men with severe OAT compared 1–2 mL intratesticular PRP injection with no treatment (control). At 3 months, significant improvements were observed in sperm concentration (mean 16.1 vs. 11.3, *p* = 0.03), motility (mean 12.0 vs. 8.9, *p* = 0.01), and a decrease in DNA fragmentation index (mean 17.2 vs. 26.6, *p* < 0.001) in the PRP group. However, normal morphology did not change significantly.^18^

In a retrospective analysis, Al‐Nasser et al.^26^ evaluated testicular sperm aspiration (TESA) samples before and after intratesticular PRP injections in 71 men with NOA. Improved spermatogenesis was observed post‐PRP injection, with 2 (2.2%) patients showing normal sperm at 2–4 months following intervention. In addition, more patients (29.7%) had few motile or immotile sperms on TESA post‐PRP compared with pre‐PRP (14.3%).

## 4 DISCUSSION

This review of human studies suggests that PRP shows promise in improving semen parameters and reducing DNA damage as a cryoprotectant, as well as improving SSR rates and the quality of retrieved sperm.

### 4.1 Cryopreservation

The main goal of cryopreservation is to preserve as many viable and normal sperm as possible post‐thaw for ART. It is known that the freezing–thawing process negatively affects sperm viability, structure and function through the formation of ice‐crystals and ROS.^4^ Hence, freeze–thaw temperature protocols, cryoprotectants and anti‐oxidants aim to mitigate these mechanisms.^4^, ^5^

Several animal studies have demonstrated that PRP can improve sperm parameters and/or reduce DNA fragmentation and enhance testicular architecture. In NOA rat models, PRP decreased markers of oxidative stress (MDA) and increased markers of spermatogenesis.^28^ Several torsion/detorsion (T/D) rat model studies have demonstrated that PRP may reverse some of the T/D adverse effects, which include improving spermatogenesis, anti‐oxidant and anti‐inflammatory activities.^29^, ^30^ Furthermore, adding PRP to semen extenders improved post‐thaw semen parameters, functionality or anti‐oxidant activity in buffalo bulls^31^, ^32^ and deer.^33^ Intratesticular injection of PRP in rabbits also improved freeze–thaw semen parameters and DNA integrity.^34^

In the past 5 years or so, the effect of PRP as a cryopreserving supplement in human semen has also been evaluated, and it has been shown in this review that PRP is an option in optimising post freeze–thaw sperm quality in men with normal^21^, ^23^, ^24^ and OAT semen samples.^20^ The potential mechanisms of PRP's effect on human semen may include mitigating oxidative stress and stabilising sperm membranes via growth factors.

### 4.2 Optimising semen parameters for assisted reproductive technology

In non‐cryopreservation scenarios, adding PRP to H_2_O_2_‐induced oxidative stress semen improved semen parameters and decreased the effect of oxidative stress compared to controls.^25^ Incubating semen from a man with asthenoteratospermia with PRP improved sperm motility and reduced DNA fragmentation, resulting in a successful ICSI outcome after two failed IVF attempts.^27^ However, more cases are required to validate the efficacy of PRP in optimising semen for ART.

### 4.3 Intratesticular injection

Intratesticular injections of PRP have been shown to improve the success rate of mTESE in men with NOA and at least one previously failed mTESE,^19^ improve sperm count and motility in men with OAT,^18^ and enhance spermatogenesis in men with NOA as seen in TESA examinations.^26^

Two studies evaluated the outcomes of ART. Gudelci et al.^19^ successfully retrieved sperm from mTESE with PRP in men who had previously failed mTESE, with an overall ICSI success rate of was 90.3% and a live birth rate of 50%. Ulhe et al.^27^ reported a case of a patient with asthenoteratospermia and two failed IVF attempts, who showed improved semen parameters following incubation with PRP. ICSI was successful and b‐HCG was positive at 14 days, suggesting pregnancy. Although these reports are promising, they lack a randomised control arm, and the results have not been replicated to date.

The only RCT included in this review assessed the effect of intratesticular PRP injection in men with OAT. Although semen parameters improved, the effect on ART outcomes remains unclear.

Again, the mechanism of PRP on testicular tissue likely involves stimulating or improving spermatogenesis, reducing oxidative stress and stabilising sperm structure.

### 4.4 Limitations

The results indicate that there is no standardised PRP protocol regarding preparation (centrifuge speed and time), optimal concentration, and volume. Additionally, the inclusion criteria varied across the studies, which included men with normal semen analysis, OAT, asthenoteratospermia, and NOA. The outcome measures also differed, encompassing semen parameters, DNA fragmentation, SSR rate, and ART success. This variation in subject inclusion and primary outcomes limited the possibility of conducting a quantitative analysis. Furthermore, not all studies included a control arm, and only one RCT was identified in this review.

### 4.5 Clinical implications

Advances in cryobiology have highlighted the potential benefits of PRP. However, it remains unclear whether PRP should be used alone or in combination with other cryoprotectants, and this requires further investigation. Regarding the improvement of semen parameters, it is still uncertain which patient groups benefit the most from PRP—whether those with asthenoteratospermia, OAT, or NOA. These findings have not been replicated in larger sample sizes or randomised settings. Additionally, the optimal PRP preparation, concentration and volume remain undefined. Future research should focus on addressing these unanswered questions, as well as exploring the role of PRP in ART.

## 5 CONCLUSION

PRP has shown promising results in improving semen parameters, cryopreservation outcomes, reducing oxidative stress and DNA fragmentation, and improving mTESE success. However, more randomised‐controlled trials with larger sample sizes are needed to replicate these findings and assess the efficacy of PRP in ART.

## AUTHOR CONTRIBUTIONS

*Conceived and/or designed the work that led to the submission; acquired data, and/or played an important role in interpreting the results*: KHP. *Drafted or revised the manuscript*: KHP. *Approved the final version*: KHP. *Agreed to be accountable for all aspects of the work in ensuring that questions related to the accuracy or integrity of any part of the work are appropriately investigated and resolved*: KHP.

## CONFLICT OF INTEREST STATEMENT

The author declares no conflicts of interest.

## Supporting information



Supporting information
