# Supplementary material for: The role and implication of platelet‐rich plasma in male factor infertility: A systematic review of human studies
Source: Andrology. 2025 Apr 17;14(1):284–93. doi: 10.1111/andr.70048 (PMC12670476; doi:10.1111/andr.70048)
Supplement: Supplementary file 1 — Supporting information [file ANDR-14-284-s001.docx]

**Supplementary Table 1.** PRISMA 2020 checklist.

| **Section and Topic** | **Item #** | **Checklist item** | **Location where item is reported** |
| --- | --- | --- | --- |
| **TITLE** | | |  |
| Title | 1 | Identify the report as a systematic review. | Title |
| **ABSTRACT** | | |  |
| Abstract | 2 | See the PRISMA 2020 for Abstracts checklist. | Abstract |
| **INTRODUCTION** | | |  |
| Rationale | 3 | Describe the rationale for the review in the context of existing knowledge. | Intro |
| Objectives | 4 | Provide an explicit statement of the objective(s) or question(s) the review addresses. | Intro |
| **METHODS** | | |  |
| Eligibility criteria | 5 | Specify the inclusion and exclusion criteria for the review and how studies were grouped for the syntheses. | SR methods section |
| Information sources | 6 | Specify all databases, registers, websites, organisations, reference lists and other sources searched or consulted to identify studies. Specify the date when each source was last searched or consulted. | SR methods section |
| Search strategy | 7 | Present the full search strategies for all databases, registers and websites, including any filters and limits used. | SR methods section |
| Selection process | 8 | Specify the methods used to decide whether a study met the inclusion criteria of the review, including how many reviewers screened each record and each report retrieved, whether they worked independently, and if applicable, details of automation tools used in the process. | SR methods section |
| Data collection process | 9 | Specify the methods used to collect data from reports, including how many reviewers collected data from each report, whether they worked independently, any processes for obtaining or confirming data from study investigators, and if applicable, details of automation tools used in the process. | SR methods section |
| Data items | 10a | List and define all outcomes for which data were sought. Specify whether all results that were compatible with each outcome domain in each study were sought (e.g. for all measures, time points, analyses), and if not, the methods used to decide which results to collect. | SR results section and results table |
|  |  |  |  |
|  |  |  |  |
|  | 10b | List and define all other variables for which data were sought (e.g. participant and intervention characteristics, funding sources). Describe any assumptions made about any missing or unclear information. | SR results section and results table |
|  |  |  |  |
|  |  |  |  |
| Study risk of bias assessment | 11 | Specify the methods used to assess risk of bias in the included studies, including details of the tool(s) used, how many reviewers assessed each study and whether they worked independently, and if applicable, details of automation tools used in the process. | SR methods section |
| Effect measures | 12 | Specify for each outcome the effect measure(s) (e.g. risk ratio, mean difference) used in the synthesis or presentation of results. | n/a |
| Synthesis methods | 13a | Describe the processes used to decide which studies were eligible for each synthesis (e.g. tabulating the study intervention characteristics and comparing against the planned groups for each synthesis (item #5)). | SR results section and results table |
|  |  |  |  |
|  |  |  |  |
|  | 13b | Describe any methods required to prepare the data for presentation or synthesis, such as handling of missing summary statistics, or data conversions. | n/a |
|  | 13c | Describe any methods used to tabulate or visually display results of individual studies and syntheses. | SR methods/results section and results table |
|  | 13d | Describe any methods used to synthesize results and provide a rationale for the choice(s). If meta-analysis was performed, describe the model(s), method(s) to identify the presence and extent of statistical heterogeneity, and software package(s) used. | n/a |
|  | 13e | Describe any methods used to explore possible causes of heterogeneity among study results (e.g. subgroup analysis, meta-regression). | n/a |
|  | 13f | Describe any sensitivity analyses conducted to assess robustness of the synthesized results. | n/a |
| Reporting bias assessment | 14 | Describe any methods used to assess risk of bias due to missing results in a synthesis (arising from reporting biases). | n/a |
| Certainty assessment | 15 | Describe any methods used to assess certainty (or confidence) in the body of evidence for an outcome. | n/a |
| **RESULTS** | | |  |
| Study selection | 16a | Describe the results of the search and selection process, from the number of records identified in the search to the number of studies included in the review, ideally using a flow diagram. | SR results section and results table |
|  |  |  |  |
|  |  |  |  |
|  | 16b | Cite studies that might appear to meet the inclusion criteria, but which were excluded, and explain why they were excluded. | PRISMA flow chart |
| Study characteristics | 17 | Cite each included study and present its characteristics. | SR results section and results table |
| Risk of bias in studies | 18 | Present assessments of risk of bias for each included study. | SR results section and Suppl table 2 |
| Results of individual studies | 19 | For all outcomes, present, for each study: (a) summary statistics for each group (where appropriate) and (b) an effect estimate and its precision (e.g. confidence/credible interval), ideally using structured tables or plots. | SR results section and results table |
| Results of syntheses | 20a | For each synthesis, briefly summarise the characteristics and risk of bias among contributing studies. | n/a |
|  | 20b | Present results of all statistical syntheses conducted. If meta-analysis was done, present for each the summary estimate and its precision (e.g. confidence/credible interval) and measures of statistical heterogeneity. If comparing groups, describe the direction of the effect. | n/a |
|  | 20c | Present results of all investigations of possible causes of heterogeneity among study results. | n/a |
|  | 20d | Present results of all sensitivity analyses conducted to assess the robustness of the synthesized results. | n/a |
| Reporting biases | 21 | Present assessments of risk of bias due to missing results (arising from reporting biases) for each synthesis assessed. | n/a |
| Certainty of evidence | 22 | Present assessments of certainty (or confidence) in the body of evidence for each outcome assessed. | n/a |
| **DISCUSSION** | | |  |
| Discussion | 23a | Provide a general interpretation of the results in the context of other evidence. | Discussion section |
|  | 23b | Discuss any limitations of the evidence included in the review. | Limitation section |
|  | 23c | Discuss any limitations of the review processes used. | Limitation section |
|  | 23d | Discuss implications of the results for practice, policy, and future research. | Discussion section |
| **OTHER INFORMATION** | | |  |
| Registration and protocol | 24a | Provide registration information for the review, including register name and registration number, or state that the review was not registered. | n/a |
|  | 24b | Indicate where the review protocol can be accessed, or state that a protocol was not prepared. | Methods |
|  | 24c | Describe and explain any amendments to information provided at registration or in the protocol. | n/a |
| Support | 25 | Describe sources of financial or non-financial support for the review, and the role of the funders or sponsors in the review. | no funding |
| Competing interests | 26 | Declare any competing interests of review authors. | no conflicts |
| Availability of data, code and other materials | 27 | Report which of the following are publicly available and where they can be found: template data collection forms; data extracted from included studies; data used for all analyses; analytic code; any other materials used in the review. | n/a |

**Supplementary Table 2.** Risk of bias assessment of included reports according to the JBI Critical Appraisal Checklist.

a) Cohort studies; b) randomized-controlled trial; c) case report.

Y, Yes; N, No; U, unclear; NA, not applicable

**a)**

| **Cohort studies** | **Gudelci et al, 2024** | **Lorian et al, 2024** | **Nabavinia et al, 2023** | **Salem et al, 2023** | **Mirzaei et al, 2022** | **Yan et al, 2021** | **Bader et al, 2020** | **Al-Nasser et al, 2018** |
| --- | --- | --- | --- | --- | --- | --- | --- | --- |
| Were the two groups similar and recruited from the same population? | NA | Y | Y | Y | Y | Y | Y | NA |
| Were the exposures measured similarly to assign people to both exposed and unexposed groups? | NA | Y | Y | Y | Y | Y | Y | NA |
| Was the exposure measured in a valid and reliable way? | Y | Y | Y | Y | Y | Y | Y | Y |
| Were confounding factors identified? | N | N | N | N | N | N | N | N |
| Were strategies to deal with confounding factors stated? | N | N | N | N | N | N | N | N |
| Were the groups/participants free of the outcome at the start of the study (or at the moment of exposure)? | Y | Y | Y | Y | Y | Y | Y | Y |
| Were the outcomes measured in a valid and reliable way? | Y | Y | Y | Y | Y | Y | Y | Y |
| Was the follow up time reported and sufficient to be long enough for outcomes to occur? | Y | Y | Y | NA | NA | Y | NA | Y |
| Was follow up complete, and if not, were the reasons to loss to follow up described and explored? | Y | NA | NA | NA | NA | NA | NA | N |
| Were strategies to address incomplete follow up utilized? | NA | NA | NA | NA | NA | NA | NA | N |
| Was appropriate statistical analysis used? | Y | Y | Y | Y | Y | Y | Y | Y |

**b)**

|  | **Randomized controlled trial** | **Fazli et al, 2024** |
| --- | --- | --- |
| 1 | Was true randomization used for assignment of participants to treatment groups? | U |
| 2 | Was allocation to treatment groups concealed? | U |
| 3 | Were treatment groups similar at the baseline? | U |
| 4 | Were participants blind to treatment assignment? | U |
| 5 | Were those delivering the treatment blind to treatment assignment? | U |
| 6 | Were treatment groups treated identically other than the intervention of interest? | Y |
| 7 | Were outcome assessors blind to treatment assignment? | U |
| 8 | Were outcomes measured in the same way for treatment groups? | Y |
| 9 | Were outcomes measured in a reliable way? | Y |
| 10 | Was follow up complete and if not, were differences between groups in terms of their follow up adequately described and analysed? | Y |
| 11 | Were participants analysed in the groups to which they were randomized? | Y |
| 12 | Was appropriate statistical analysis used? | Y |
| 13 | Was the trial design appropriate and any deviations from the standard RCT design (individual randomization, parallel groups) accounted for in the conduct and analysis of the trial? | Y |

**c)**

|  | **Case report** | **Ulhe et al, 2024** |
| --- | --- | --- |
| 1 | Were patient’s demographic characteristics clearly described? | Y |
| 2 | Was the patient’s history clearly described and presented as a timeline? | Y |
| 3 | Was the current clinical condition of the patient on presentation clearly described? | Y |
| 4 | Were diagnostic tests or assessment methods and the results clearly described? | Y |
| 5 | Was the intervention(s) or treatment procedure(s) clearly described? | N |
| 6 | Was the post-intervention clinical condition clearly described? | Y |
| 7 | Were adverse events (harms) or unanticipated events identified and described? | N |
| 8 | Does the case report provide takeaway lessons? | Y |
